# Attaques de requins en Nouvelle-Calédonie de 1958 à 2020 : revue de cas

**DOI:** 10.48327/mtsi.v2i1.2022.209

**Published:** 2022-02-10

**Authors:** Claude MAILLAUD, Philippe TIRARD, Philippe BORSA, Anne-Laure GUITTONNEAU, Joseph FOURNIER, Mohammed NOUR

**Affiliations:** 1 ISEA (Institut de sciences exactes et appliquées) (EA 7484), Université de la Nouvelle-Calédonie, BP R4, 98851 Nouméa, Nouvelle-Calédonie; 2 9 rue Émile Legrand - Anse Vata, Nouméa, 98800 Nouméa, Nouvelle-Calédonie; 3 Institut de recherche pour le développement, IRD-UMR 250 « Écologie marine tropicale des océans Pacifique et Indien », Centre IRD Occitanie, 911 avenue Agropolis, 34394 Montpellier Cedex 5, France; 4 SAMU/SMUR/SAU/UHCD, Centre hospitalier territorial de Nouvelle-Calédonie, BP J5, 98849 Nouméa, Nouvelle-Calédonie; 5 Service de Chirurgie orthopédique et traumatologique, Centre hospitalier territorial de Nouvelle-Calédonie, BP J5, 98849 Nouméa, Nouvelle-Calédonie

**Keywords:** Attaque de requin, Stimulation alimentaire, Chasse sous-marine, Requin tigre, Requin bouledogue, Nouvelle-Calédonie, Océan pacifique, Shark attack, Feeding stimulus, Spearfishing, Tiger shark, Bull shark, New Caledonia, Pacific Ocean

## Abstract

**Contexte et objectifs:**

Nous présentons une série de cas d’attaques de requins survenues en Nouvelle-Calédonie, afin de proposer aux usagers du milieu marin concernés et aux autorités en charge de la sécurité du public un état des lieux de cette problématique.

**Méthodes:**

Un recueil de cas réalisé par les auteurs a fait l’objet d’une base de données, dont les résultats sont analysés et présentés ici sous forme synthétique.

**Résultats:**

Nous avons répertorié 67 cas d’attaques de requins en Nouvelle-Calédonie de 1958 à 2020, dont 13 fatales. Le nombre des attaques a augmenté au fil des années. Les attaques ont concerné majoritairement des chasseurs sous-marins et des apnéistes récoltant des invertébrés (58,5 % du total des attaques). La proportion d’attaques de baigneurs, nageurs et adeptes du snorkeling (18,5 % du total des attaques) et celle des pratiquants des sports de glisse (14 %) pourraient avoir augmenté ces dernières décennies. Une attaque de plongeur en scaphandre autonome a été répertoriée. La létalité des attaques est de près d’une sur cinq, supérieure à la moyenne mondiale. Les principales espèces en cause sont le requin tigre *Galeocerdo cuvier* (20 attaques, dont 8 mortelles) et le requin bouledogue *Carcharhinus leucas* (14 attaques, dont 2 mortelles). Deux attaques ont été attribuées au grand requin blanc *Carcharodon carcharías,* dont une mortelle.

**Conclusions:**

La stimulation alimentaire apparaît comme le principal facteur favorisant les attaques. L’information du public mériterait d’être optimisée dans une perspective de prévention des accidents.

## Abstract (en)

**Background and objectives:**

Recent shark attacks in New Caledonia have prompted local authorities to elaborate a risk-management plan. The objective of the present paper is to produce detailed data on shark attacks that occurred in New Caledonian waters for the last few decades, as well as on the injuries of the victims, in order to inform rescue and medical services as well as authorities in charge of educating the public and providing security.

**Methods:**

Incidents involving sharks and humans in New Caledonia for the last six decades were included into a database. Sharks were tentatively identified to species according to the shape, size and other external characteristics of injuries to the victims, together with witness accounts. The severity of shark bites was evaluated against the scale proposed by A.K. Lentz and co-authors (Am Surg. 2010;76:101-6).

**Results:**

Sixty-seven shark-attack cases were recorded in New Caledonia from 1958 to 2020, of which 13 were lethal. The majority of the attacks concerned spearfishers and freedivers collecting invertebrates (58.5% of total). In the last decades, shark attacks may have increased towards bathers, swimmers and snorkelers (18.5%), and people taking part in water sports including surf, kitesurf, windsurf and SUP foil (14%). One scuba diver was also attacked (1.5%). Twenty attacks including 8 lethal ones were ascribed to the tiger shark *Galeocerdo cuvier;* 14 attacks including 2 lethal ones to the bull shark *Carcharhinus leucas;* 2 attacks including 1 lethal to the great white shark *Carcharodon carcharías.* The lethality of attacks was almost one in five, above the global average.

**Conclusions:**

Feeding incentive appeared to be a frequent factor triggering attacks. The education of the public should be promoted as a preventive measure aiming to reduce the risk of such accidents.

## Introduction

**Figure 1:**
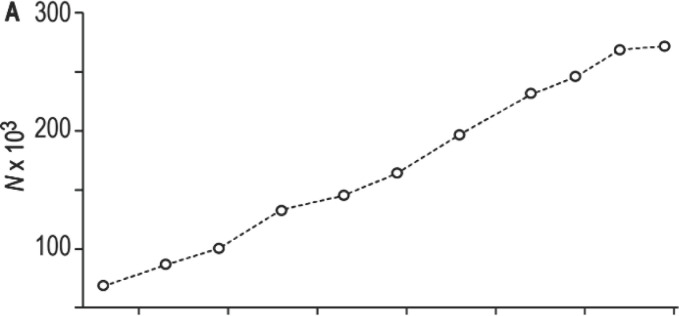
Nouvelle-Calédonie. Évolution démographique humaine au cours de sept dernières décennies (1955-2020); chaque cercle correspond à un recensement de la population New Caledonia. Human demographic evolution over the last seven decades (1955-2020); each circle corresponds to a population census.

La Nouvelle-Calédonie, ensemble insulaire français du Pacifique Sud, bénéficie d’un climat tropical, de 3 367 km de linéaire côtier, d’une barrière corallienne de 1 600 km de long délimitant un lagon d’environ 24 000 km^2^ d’une profondeur moyenne de 25 m et de récifs coralliens couvrant environ 8 000 km^2^ [7, 27, 44]. À l’exception des toutes dernières années, la Nouvelle-Calédonie a vu sa démographie progresser régulièrement du fait d’un excédent naturel et d’un solde migratoire positif: au cours des 6 dernières décennies, la population du territoire est passée de moins de 70 000 habitants (en 1956) à plus de 270 000 (271 407 habitants en 2019) [24] (Fig. 1). L’agglomération principale de Nouméa et les communes adjacentes concentrent les deux tiers de la population. Outre la pêche, pratiquée traditionnellement, de nombreuses activités nautiques s’offrent aux habitants des zones littorales: baignade, chasse et plongée sous-marines, apnée, voile, planche à voile, kitesurf, stand up paddle (SUP), kayak et va’a (pirogue polynésienne), jet-ski, etc.

Parmi la trentaine d’espèces de requins considérées comme dangereuses ou potentiellement dangereuses pour l’homme, une vingtaine sont répertoriées en Nouvelle-Calédonie:

*Alopias superciliosus, A. vulpinus, Carcharhinus albimarginatus, C. amblyrhynchos, C. brevipinna, C. falciformis, C. leucas, C. limbatus, C. longimanus, C. melanopterus, C. obscurus, C. plumbeus, C. sorrah, Carcharodon carcharias, Galeocerdo cuvier, Isurus oxyrinchus, Nebrius ferrugineus, Negaprion acutidens, Prionace glauca, Sphyrna lewini, S. mokarran et Triaenodon obesus* [2, 18, 23, 28, 32, 46, 51] (Tableau II pour les noms vernaculaires).

Le risque de rencontre, voire d’attaque, apparaît dépendant des activités humaines, possiblement du fait de l’augmentation de la fréquentation du lagon [9, 37]. De récentes attaques, pour certaines aux abords de Nouméa, sont à l’origine d’une préoccupation des autorités locales vis-à-vis de ce type d’accident, laquelle se traduit par une volonté de définir un plan de « gestion » du risque [1].

L’objectif du présent travail est de produire des données chiffrées détaillées sur les attaques survenues ces dernières décennies dans les eaux calédoniennes, ainsi que sur les traumatismes infligés aux victimes. Le but est de contribuer à l’information des personnels en charge des secours aux victimes (maîtres-nageurs sauveteurs, sapeurs-pompiers, personnels des services d’urgence mobiles, ainsi qu’urgentistes, chirurgiens et réanimateurs hospitaliers) et d’orienter les décisions des représentants institutionnels en charge de la sécurité du public.

## Méthodes

Nous proposons une revue systématique de l’ensemble des cas d’attaques de requins relevés en Nouvelle-Calédonie de 1958 à 2020. Notre analyse porte sur trois séries de cas dont les données ont été fusionnées: la première série de cas a été relevée par F. Dreyer [14], au cours d’une enquête rétrospective et prospective portant sur la période de 1958 à 2001, menée au Centre hospitalier territorial de Nouvelle-Calédonie (CHT-NC) entre mai 2000 et avril 2001, en collaboration avec l’un des auteurs (P.T.). La deuxième série de cas est l’aboutissement d’une enquête prospective menée de mai 2002 à avril 2007 au CHT-NC par l’un des auteurs (C.M.); elle a été complétée par des données rétrospectives tirées essentiellement de questionnaires adressés aux médecins des dispensaires de Brousse et des Îles courant 2006, portant sur la période de 1958 à 2007. Ces deux premières séries ont fait l’objet d’une publication de deux des auteurs en 2009 (C.M., P.T.) [35]. La troisième résulte d’un travail prospectif mené de mai 2007 à décembre 2020 par trois des auteurs (P.T., C.M., A-L.G.), auxquels un quatrième s’est associé en 2020 (J.F.), sur la base d’informations issues du CHT-NC, ou recueillies auprès de différentes sources (médias, forces de l’ordre, victimes elles-mêmes). Le présent travail constitue donc une mise à jour des données publiées précédemment à partir des deux premières séries, soit 40 cas, de 1958 à 2007 [35].

Nous avons retenu comme définition d’une attaque de requin celle donnée précédemment par G. Van Grevelynghe à La Réunion, inspirée de celle de G. Cliff du KwaZulu Natal Shark Board: « Une attaque est un accident au cours duquel un contact physique agressif a été formellement établi entre un ou des requins et une ou des victimes humaines vivantes, ayant entraîné des blessures ou le décès de la ou des victimes, ou une détérioration importante d’équipements (morsure de planche, arrachage de palme). Cette définition exclut du recensement les attaques sans blessure ni dégradation matérielle (cas de plongeurs chargés par des requins mais qui purent sortir de l’eau indemnes, cas d’attaques d’embarcations sans dommage, et surtout cas de prédations posthumes: cadavres de noyés dévorés secondairement) » [10,49,50,51]. Nous avons inclus dans le présent travail les attaques n’ayant pas occasionné de blessures sur un humain, mais à l’origine de dommages matériels, dont les morsures de planches. Nous n’avons pas retenu les attaques d’embarcations à moteur. Nous avons exclu quelques cas où une séquence d’événements a conduit à une présomption élevée d’attaque mortelle, mais où l’absence de dépouille et de témoins ne nous a pas formellement permis d’éliminer l’hypothèse d’une prédation posthume. Nous avons ainsi exclu de notre recueil des cas de disparition en mer, pour certains récents, y compris lorsque des traces de morsures ont été mises en évidence sur le matériel de la victime, dès lors que le corps de celle-ci n’a pas été retrouvé et qu’il n’y a pas eu de témoignage d’une attaque. Nous n’avons pas opéré de discrimination, à quelques exceptions près, entre attaques dites provoquées et non provoquées en raison du caractère ténu, voire, à nos yeux, artificiel, de la distinction entre les deux.

**Figure 2:**
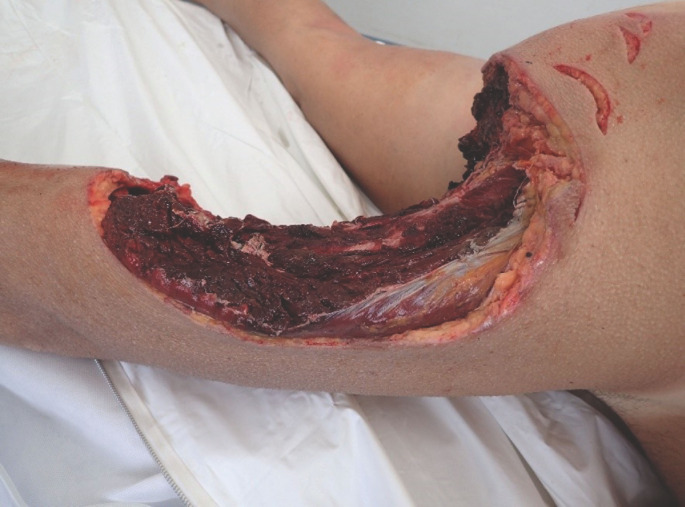
Morsure létale de requin tigre (crédit Claude Maillaud) Fatal tiger shark bite (credit Claude Maillaud)

L’identification des requins en cause dans les attaques a été effectuée en comparant les lésions constatées chez les victimes ou les marques imprimées sur leur équipement avec une base de données morphométriques (dentures de spécimens identifiés et mesurés) établie par l’un des auteurs (P.T.) et permettant d’estimer espèce et taille du requin à partir de ses empreintes dentaires [46, 47]. Cette évaluation a été réalisée soit après examen de la victime par l’un, l’autre ou deux des auteurs, (P.T., C.M.), l’un ancien plongeur biologiste à l’IRD, l’autre médecin légiste, soit après examen des lésions sur documents photographiques par P.T. et C.M., ces données étant recoupées le cas échéant avec les descriptions de l’animal rapportées par les victimes ou les témoins de l’accident au cours d’entretiens destinés à recueillir également les circonstances de ceux-ci, réalisés par P.T. ou C.M. (Fig. 2).

Enfin, pour catégoriser la gravité des lésions, nous avons utilisé le shark-induced trauma (SIT) scale [30] dont nous proposons une traduction française (Tableau I), de préférence à la classification de Durban [11]. Le SIT Scale est une grille d’évaluation prenant en compte, outre les lésions vasculaires, le retentissement de celles-ci, à savoir la présence ou non d’un choc hémorragique conditionnant à court terme le pronostic vital, les lésions musculaires, tendineuses, nerveuses et osseuses en incluant le niveau de complexité de leur traitement, ainsi que l’existence prévisible ou établie de séquelles fonctionnelles. En revanche, la classification de Durban, selon laquelle le pronostic vital est strictement corrélé au nombre et au calibre des vaisseaux lésés chez la victime, nous est apparue moins pertinente dans le contexte océanien. En effet, s’agissant d’accidents survenant dans des ensembles géographiques éclatés, les délais de prise en charge et la qualité de celle-ci nous sont apparus déterminants dans la survie des victimes de traumatismes graves, le pronostic vital étant susceptible d’être engagé indépendamment du seul calibre des vaisseaux lésés [33].

**Tableau I:** Attaques de requins sur des humains: échelle de gravité des lésions (SIT Scale, d’après [30]) Shark attacks on humans: scale of trauma severity as adapted from the SIT Scale of [30]

Niveau de gravité	Lésions constatées
1 (L1)	Cutanées et sous-cutanées
2 (L2)	Cutanées, sous-cutanées, musculaires, tendineuses, osseuses, sans répercussion hémodynamique ni séquelle fonctionnelle
3 (L3)	Cutanées complexes, sous-cutanées, musculaires, tendineuses et osseuses, avec spoliation sanguine et mise en jeu du pronostic fonctionnel
4 (L4)	Atteinte des tissus profonds, perte de fonction d’une extrémité ou d’un organe, atteinte d’un gros vaisseau et mise en jeu immédiate du pronostic vital
5 (L5)	Le plus souvent fatales de par leur situation et la survenue d’une spoliation sanguine massive

## Résultats

### Distribution temporelle et spatiale des attaques

**Figure 3:**
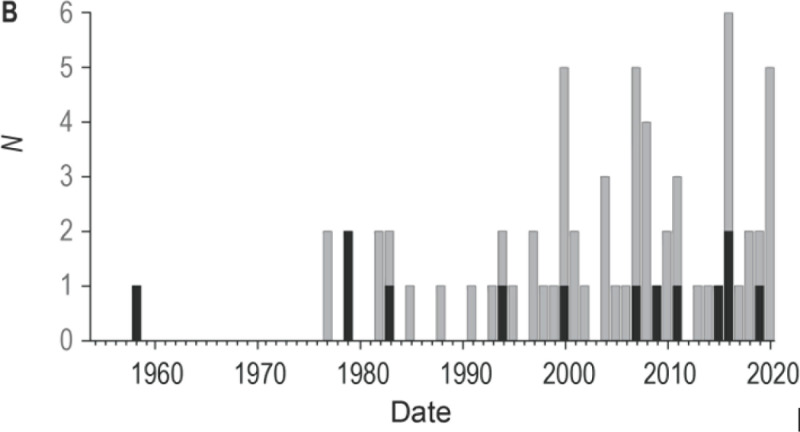
Nouvelle-Calédonie. Fréquence annuelle des cas connus d’attaques de requins au cours de sept dernières décennies (1955-2020); en grisé: attaques non létales; en noir: attaques létales New Caledonia. Frequency of known cases of shark attacks by year over the last seven decades (1955-2020); grey: non-lethal attacks; black: lethal attacks

Nous avons répertorié 67 attaques de requins en Nouvelle-Calédonie dont 13 attaques mortelles entre 1958 et 2020, soit sur 63 ans (Fig. 3). Les données ont été déposées dans la base de données DataSuds de l’IRD [13, 34]. La fréquence de ce type d’accident est d’un peu plus de 1 par an. Toutefois, leur répartition au fil de ces 6 décennies est hétérogène: 5 attaques dont 3 mortelles de 1958 à 1980, 20 dont 3 mortelles de 1981 à 2000, 42 dont 7 mortelles de 2001 à 2020. La fréquence annuelle des attaques recensées dans notre base de données augmente de façon significative: de 0,22 par an durant la première période, elle passe à 1 par an durant la seconde et dépasse 2 par an durant la période la plus récente (test de Kruskal-Wallis: P < 0,001). La létalité des attaques est globalement de 19 %.

**Figure 4:**
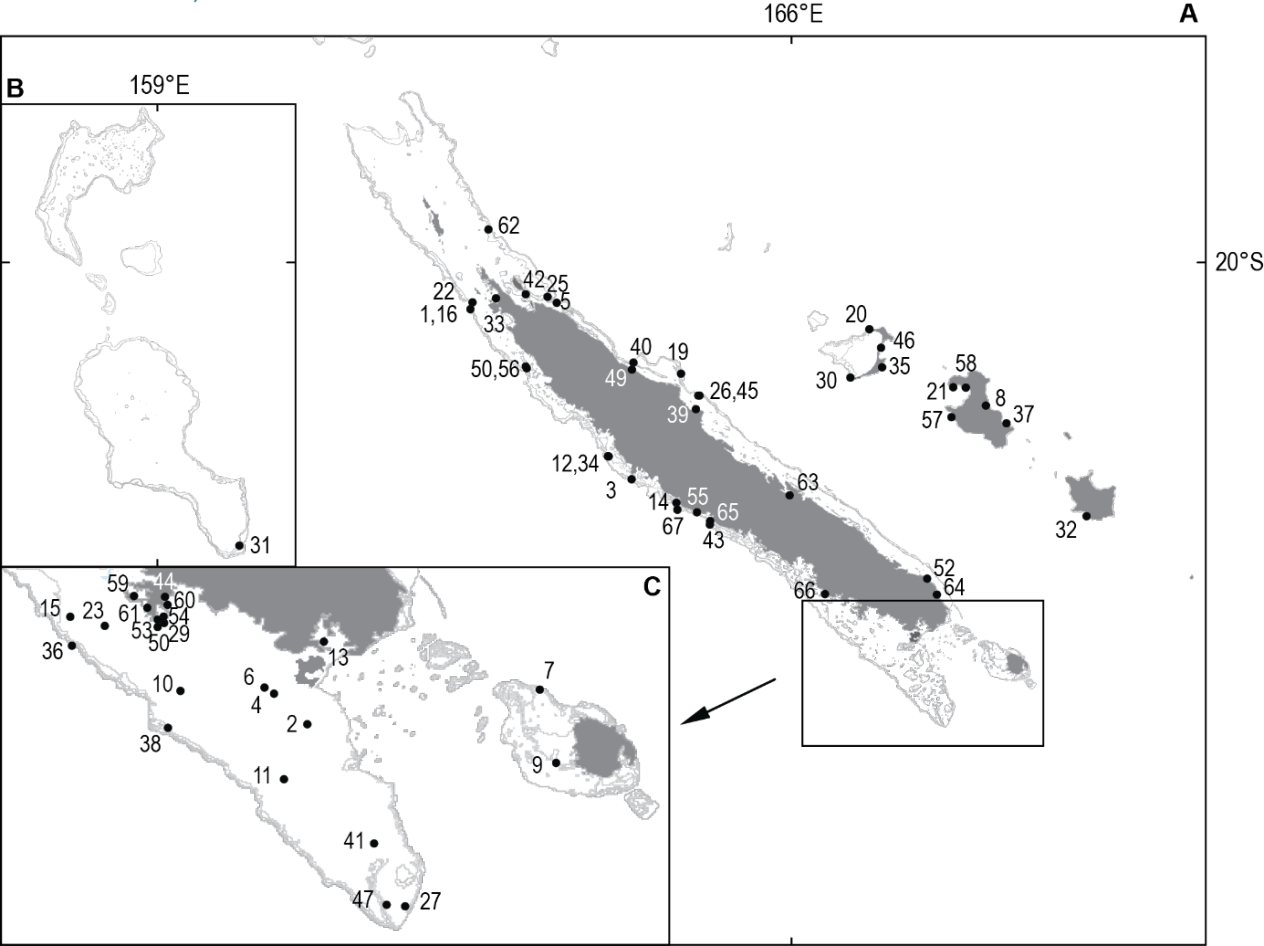
Geographic distribution of shark attacks in New Caledonia; each black dot represents the site of one or several attacks; associated numbers are those of the chronological list of attacks in [34]. A. New Caledonia and Loyalty Islands. B. Chesterfield-Bampton and Bellona Atolls. C. Southern lagoon of New Caledonia, enlarged. Distribution géographique des attaques de requins en Nouvelle-Calédonie; chaque point noir représente un site d’attaque; le numéro associé à chaque point renvoie à la liste chronologique des attaques répertoriées par [34]. A. Nouvelle-Calédonie et Îles Loyauté. B. Atolls Chesterfield-Bampton et Bellona. C. Agrandissement du lagon sud de la Nouvelle-Calédonie.

La distribution géographique des attaques (Fig. 4) est la suivante: 50 cas sur 67 (soit 74,5 %) dans le lagon (comprenant l’île Ouen) ou sur le récif de la Grande Terre, 4 cas (6 %) à l’Île des Pins, 11 cas (16,5 %) aux Îles Loyauté (Lifou: 6 cas; Ouvéa: 4 cas; Maré: 1 cas), 2 cas (3 %) aux Îles Bélep et aux Îles Chesterfield. Parmi les attaques survenues sur la Grande Terre, 8 (12 %) ont eu lieu dans les eaux de Nouméa depuis 2010.

**Figure 5:**
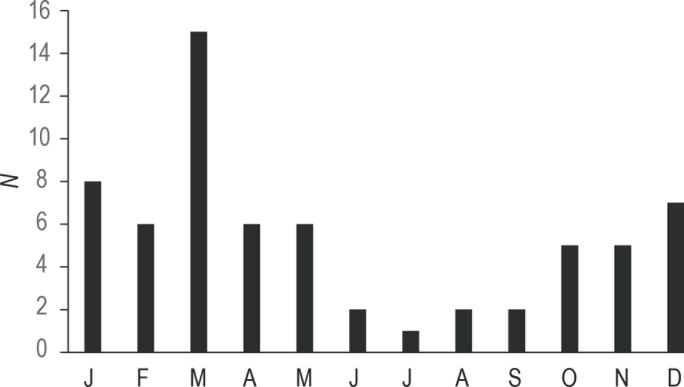
Fréquence mensuelle des attaques de requins documentées en Nouvelle-Calédonie de 1958 à 2020 (N = 65) Monthly frequency of shark attacks in New Caledonia, 1958-2020 (N = 65)

La date exacte de l’attaque est connue dans 65 cas. Une variation saisonnière de l’occurrence des accidents est notée, ceux-ci survenant principalement durant l’été austral, soit de décembre à mars, avec un pic au mois de mars (Fig. 5). Les victimes sont de sexe masculin dans 61 cas (91 %). L’âge des victimes est connu dans 65 cas; il est en moyenne de 32 ans avec des extrêmes de 10 à 69 ans.

### Activités pratiquées par les victimes lors des attaques

**Figure 6:**
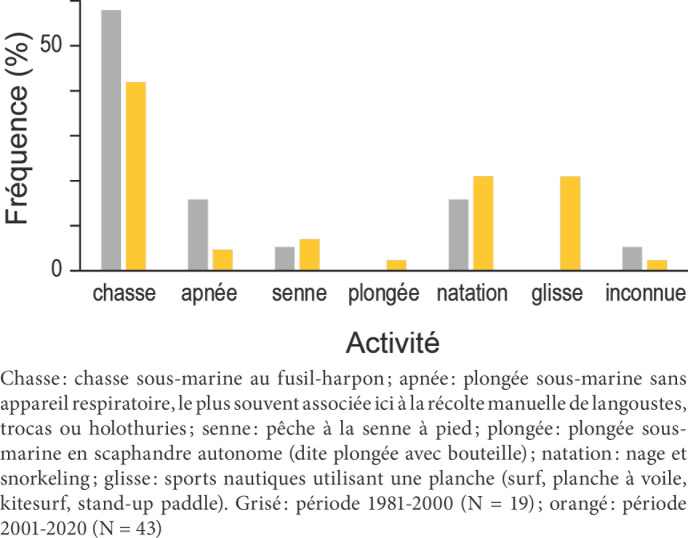
Distribution des victimes d’attaques en Nouvelle-Calédonie par catégorie d’usage du milieu marin, sur les quatre dernières décennies Distribution of shark-attack victims in New Caledonia by category of maritime activity across the last four decades

Les victimes des attaques sont des pratiquants des activités suivantes: (i) chasse sous-marine dans 32 cas (49 %); (ii) apnée dans 7 cas (11 %), dont pêche au troca (gastéropode exploité pour sa chair et pour sa nacre, dont la coquille en forme de cône peut atteindre 15 cm de diamètre à sa base) dans 3 cas; pêche à la langouste dans 2 cas; pêche aux holothuries dans 1 cas; apnée sans activité de prélèvement dans 1 cas; au total, chasseurs sous-marins et apnéistes récoltant des invertébrés représentent 38 cas (58,5 %); (iii) baignade, natation ou snorkeling (palmes-masque-tuba, ou PMT) dans 12 cas (18,5 %); (iv) plongée sous-marine en scaphandre autonome dans 1 cas (1,5 %); (v) sports de glisse dans 9 cas (14 %), dont kitesurf dans 5 cas, surf dans 2 cas, planche à voile dans 1 cas, SUP foil dans 1 cas; pêche à la senne dans 4 cas (6 %). L’activité n’est pas connue dans 2 cas. Parmi les chasseurs sous-marins, 25 (78 % d’entre eux) avaient capturé ou blessé au moins un poisson avant l’attaque. Dans cette catégorie d’usagers, trois ont eu un comportement pouvant être considéré comme susceptible d’avoir fortement favorisé, si ce n’est provoqué, l’attaque: deux ont décoché une flèche à un requin à haute dorsale *Carcharhinus plumbeus* inquisiteur; le troisième a délibérément saisi par la caudale un requin nourrice *Nebrius ferrugineus* ; parmi les nageurs, l’un en PMT a pratiqué le Chasse: chasse sous-marine au fusil-harpon; apnée: plongée sous-marine sans appareil respiratoire, le plus souvent associée ici à la récolte manuelle de langoustes, trocas ou holothuries; senne: pêche à la senne à pied; plongée: plongée sous-marine en scaphandre autonome (dite plongée avec bouteille); natation: nage et snorkeling; glisse: sports nautiques utilisant une planche (surf, planche à voile, kitesurf, stand-up paddle). Grisé: période 1981-2000 (N = 19); orangé: période 2001-2020 (N = 43) nourrissage de requins, activité considérée comme à risque élevé d’attaque. Au total, 52 victimes étaient entièrement immergées lors de l’attaque (80 %), alors que 13 (20 %) l’étaient partiellement ou évoluaient au-dessus de la surface. Une augmentation relative du nombre d’attaques sur des nageurs et des pratiquants des sports de glisse est possible (Fig. 6) mais celle-ci n’est pas significative (test de Chi-2: P = 0,094).

Les victimes des attaques létales sont des pratiquants des activités suivantes: chasse sous-marine et apnée avec activité de capture (troca, langouste, holothurie) dans 7 cas (soit 54 % des décès); natation, avec ou sans PMT dans 3 cas (23 %), un sport de glisse dans 3 cas (23 %) dont kitesurf (2 cas) et surf (1 cas). Alors que jusqu’en 2009, apnéistes et chasseurs sous-marins représentaient 100 % des victimes d’attaques létales en Nouvelle-Calédonie [35], cette proportion est passée à 54 % fin 2009, les 46 % restant étant également répartis entre, d’une part, les baigneurs, nageurs et adeptes du snorkeling, et, d’autre part, les pratiquants des sports de glisse.

### Facteurs favorisants identifiés

La capture d’animaux marins et en particulier de poissons au fusil sous-marin est génératrice de stimuli auxquels les requins sont sensibles [8, 20, 39]. Par ordre de distance décroissante, il peut s’agir de stimuli acoustiques (bruits, vibrations), olfactifs (par ex. consécutifs au saignement de la proie), visuels (par ex. la proie se débattant en surface), et électriques et thermiques (émis par la personne ou la proie et détectés par les ampoules de Lorenzini, lesquelles sont des organes sensoriels présents chez les requins, les raies et les chimères, leur permettant de détecter champs électromagnétiques et gradients de température). Les rejets d’activités de pêche, les restes alimentaires, les carcasses d’animaux, les effluves en provenance de zones littorales représentent de tels stimuli olfactifs, en particulier pour les grands requins prédateurs opportunistes. Nous avons regroupé ces différents types de stimuli sous la dénomination de « stimulation alimentaire », bien que celle-ci recouvre des phénomènes variés.

Une stimulation alimentaire est établie lorsqu’un poisson ou une langouste a été capturé(e) ou blessé(e) par un chasseur sous-marin. Nous ignorons si elle existe s’agissant des trocas et des holothuries mais des stimuli olfactifs sont possibles. La présence d’une stimulation alimentaire, probablement olfactive, a par ailleurs été suspectée dans plusieurs autres cas: 1 nageuse en PMT a subi une attaque mortelle sur un site où les reliefs alimentaires d’une fête avaient été rejetés à la mer [47]; 1 pêcheur à la senne a été mordu non loin d’une carcasse de cachalot échouée sur la côte; 2 kitesurfers ont été tués dans, ou à relative proximité, d’un chenal menant à un port thonier; 1 nageuse est décédée des suites d’une attaque au bord d’une plage où le ruissellement des eaux pluviales a pu entraîner jusqu’au lagon des produits de la décomposition de carcasses de cerf abattus par des chasseurs, ainsi que des effluves d’engrais de poisson utilisé à proximité du rivage. Au total, une stimulation alimentaire résultant le plus souvent d’activités humaines apparaît comme un facteur d’attaque documenté ou probable dans 32 cas (49 %) parmi ceux relevés dans cette série. S’y ajoutent 2 attaques récentes à Nouméa, attribuées à des requins bouledogues *Carcharhinus leucas,* dont la présence sur la côte est susceptible d’avoir été favorisée par des rejets issus de la pêche ainsi que des rejets organiques produits par la population humaine.

Parmi les autres facteurs d’attaque de requin classiquement identifiés au niveau mondial [10, 11, 32, 46, 49, 50, 51], une eau trouble ou la présence de la victime dans l’eau la nuit ou au crépuscule a été notée dans 17 cas (25 %). La visibilité était considérée comme moyenne à très bonne dans la majorité des autres cas. L’isolement des victimes n’apparaît pas comme un facteur déterminant des attaques, bien que cette donnée apparaisse peu exploitable dans cette série du fait de la forte incertitude quant à l’éloignement des chasseurs sous-marins les uns des autres dans la plupart de ces cas.

Les accidents sont survenus en surface 52 fois (86 %), lors de la remontée en fin d’apnée ou à proximité du fond à des profondeurs comprises entre 5 et 15 mètres dans 7 cas (12 %), à grande profondeur (36 m) dans 1 cas (2 %), cette donnée n’étant pas renseignée dans 7 cas.

### Espèces incriminées

L’identification de l’espèce en cause dans les attaques peut être proposée dans 53 cas sur 67. Parmi les espèces non identifiées (les 14 cas restants), l’analyse des lésions permet de présumer qu’il s’agit majoritairement de *Carcharhinidae* des genres *Carcharhinus* ou *Negaprion* de taille moyenne. Parmi les responsables d’attaques identifiés à l’espèce, ont été mis en cause: le requin tigre *Galeocerdo cuvier* 20 fois (38 %), le requin bouledogue *(Carcharhinus leucas)* 14 fois (26 %), le requin gris de récif *C. amblyrhynchos* 8 fois (15 %), le requin citron *N. acutidens* 3 fois (6 %), le grand requin blanc *Carcharodon carcharias* 2 fois (4 %), le requin à pointes blanches de récif *Carcharhinus albimarginatus* 2 fois (4 %), le requin gris à haute nageoire dorsale *C. plumbeus* 2 fois (4 %), le grand requin marteau *Sphyrna mokarran* 1 fois (2 %) et le requin nourrice ou requin dormeur *Nebrius ferrugineus* 1 fois (2 %).

Les attaques mortelles, au nombre de 13, ont été le fait du requin tigre 8 fois (ce qui représente 73 % de celles-ci), du requin bouledogue 2 fois (18 %), du grand requin blanc 1 fois (9 %), l’espèce étant non identifiée dans 2 cas; toutefois, les caractéristiques de l’un des 2 cas semblent désigner une attaque par le requin citron. La létalité des attaques par requin tigre est de 40 % (8 décès pour 20 attaques). Nous proposons un tableau récapitulatif du nombre d’attaques et du nombre d’attaques mortelles relevés pour chaque espèce (Tableau II).

**Tableau II:** Espèces de requins impliqués dans les attaques et dans les attaques létales sur des humains Shark species involved in attacks and in lethal attacks on humans

Espèce		Nombre d’attaques	Attaques létales
Nom scientifique	Nom vernaculaire		
*Galeocerdo cuvier* (Fig. 7)	Requin tigre	20	8
*Carcharhinus leucas* (Fig. 8)	Requin bouledogue	14	2
*Carcharodon carcharias* (Fig. 9)	Grand requin blanc	2	1
*Carcharhinus amblyrhynchos* (Fig. 10)	Requin gris de récif	8	_-_
*Negaprion acutidens* (Fig. 11)	Requin citron	3	_-_
*C. albimarginatus* (Fig. 12)	Requin pointe blanche de récif	2	_-_
*C. plumbeus*	Requin gris à haute nageoire dorsale	2	_-_
*Sphyrna mokarran*	Grand requin marteau	1	_-_
*Nebrius ferrugineus*	Requin nourrice	1	_-_
Non identifiée	Non identifiée	14	2
**Total**		**67**	**13**

**Figure 7:**
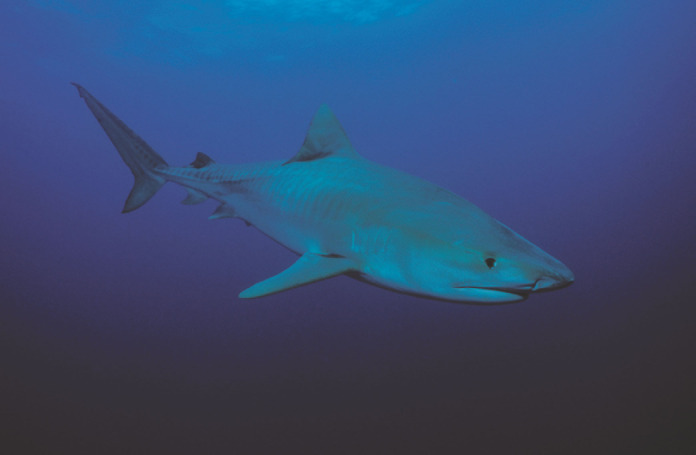
Requin tigre *Galeocerdo cuvier* *Tiger shark* Galeocerdo cuvier

**Figure 8:**
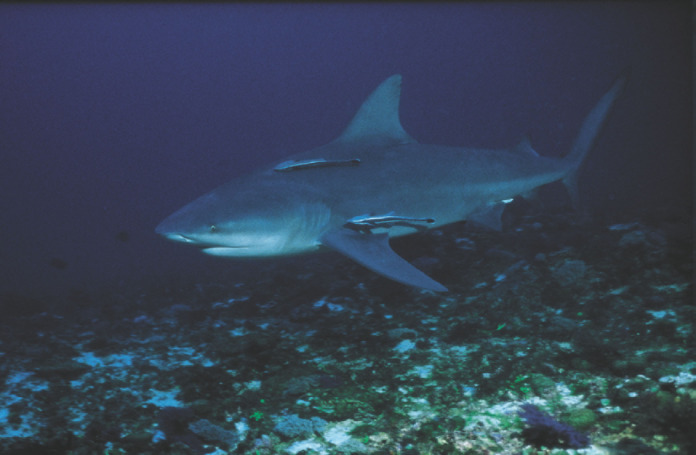
Requin bouledogue *Carcharhinus leucas* *Bull shark* Carcharhinus leucas

**Figure 9:**
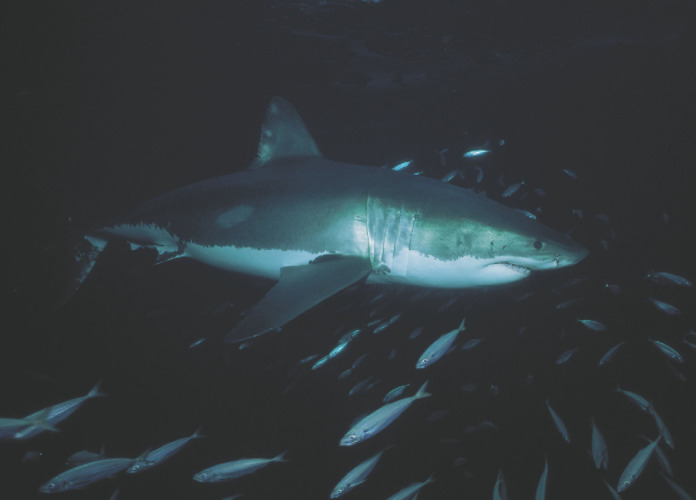
Grand requin blanc *Carcharodon carcharias* *Great white shark* Carcharodon carcharias

**Figure 10:**
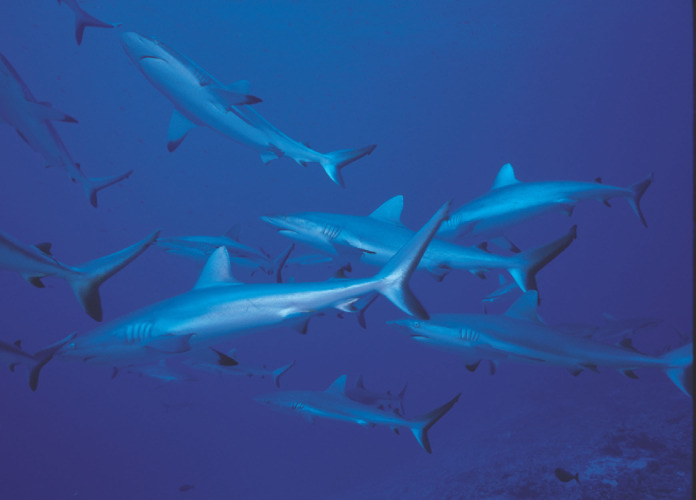
Banc de requins gris de récif *Carcharhinus amblyrhynchos* *School of grey reef sharks* Carcharhinus amblyrhynchos

**Figure 11:**
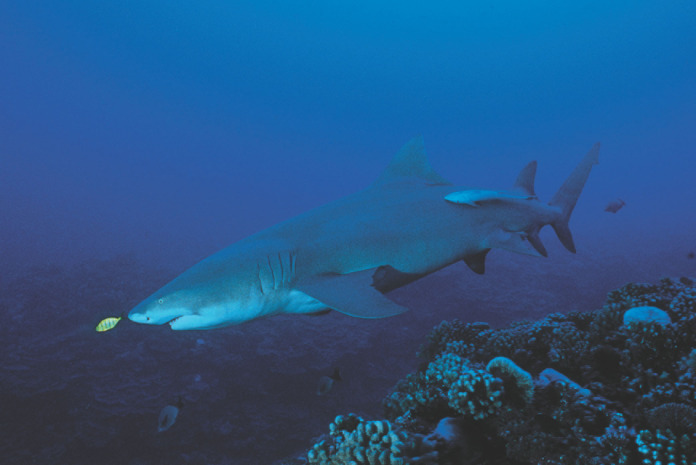
Requin citron *Negaprion acutidens* *Sicklefin lemon shark* Negaprion acutidens

**Figure 12:**
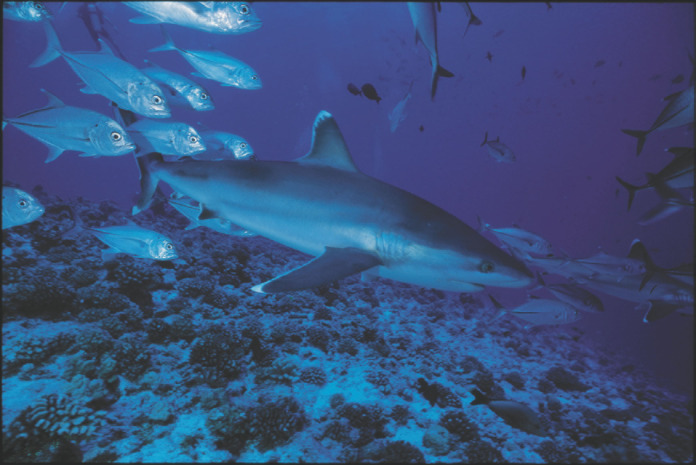
Requin pointe blanche de récif *Carcharhinus albimarginatus* *Silvertip shark* Carcharhinus albimarginatus

Les attaques ayant eu lieu aux abords de Nouméa sont imputées au requin bouledogue dans 4 cas, au requin tigre dans 2 cas, à une espèce indéterminée dans 1 cas. Elles ont concerné des adeptes des sports de glisse dans 5 cas (kitesurf: 3 cas; planche à voile: 1 cas; SUP foil: 1 cas), ainsi que 1 baigneur et 2 chasseurs sous-marins. Les 2 dernières attaques ont causé des lésions sévères, avec mise en jeu du pronostic vital et séquelles fonctionnelles importantes.

La taille de l’animal en cause a été estimée à plus de 2 m dans 43 cas (soit 72 % de ceux-ci), comprise entre 1,50 et 2 m dans 12 cas (20 %) et inférieure à 1,50 m dans 4 cas (7 %). Elle est indéterminée dans 7 cas.

### Gravité des lésions

Dans 3 cas (5 % des attaques), il n’a pas été constaté de lésion physique, mais uniquement la détérioration du matériel de la victime. Parmi les 63 cas d’attaques avec blessures physiques, les lésions observées se situent sur les membres inférieurs dans 43 cas (68 %), les membres supérieurs dans 22 cas (34 %), le tronc dans 9 cas (14 %) et la tête et/ou le cou dans 5 cas (8 %); les lésions sont multiples (plus d’un siège ou plus d’un membre) dans 16 cas (25 %).

Le SIT Scale utilisé pour évaluer la gravité des lésions ne prend pas en compte les détériorations de matériel, dont nous avons relevé 3 cas. Nous présentons les pourcentages suivants pour les 64 cas de victimes de lésions physiques. Par ordre de sévérité décroissante, nous avons répertorié 14 victimes de lésions de niveau 5 (soit 22 % des blessés), 2 victimes de lésions de niveau 4 (3 %), 11 victimes de lésions de niveau 3 (17 %), 16 victimes de lésions de niveau 2 (25 %), 21 victimes de lésions de niveau 1 (33 %). Les lésions des victimes décédées étaient toutes de niveau 5.

## Discussion

L’incidence des attaques de requins dans le monde en 2020 a été estimée par l’International Shark Attack File (ISAF) à une centaine [26]. Cet organisme distingue les attaques « non provoquées » des attaques « provoquées », les secondes comprenant toutes celles au cours desquelles la victime est considérée comme ayant eu un comportement à risque d’accident, tel que la pratique de la chasse sous-marine ou le nourrissage de requins. En 2020, l’ISAF a recensé 57 attaques « non provoquées » et 39 « provoquées ». S’agissant des années antérieures, seules sont accessibles les données relatives aux attaques non provoquées, ce qui rend délicates les comparaisons avec notre série de cas, laquelle ne suit pas cette distinction.

Globalement, l’incidence des attaques de requins non provoquées dans le monde est estimée par l’ISAF à moins d’une centaine par an [26]. De 1580 à 2020, les nombres de cas cumulés les plus élevés ont été enregistrés aux États-Unis d’Amérique (1 516), en Australie (670), en République Sud-Africaine (255), au Brésil (107), en Nouvelle-Zélande (53), en Papouasie-Nouvelle-Guinée (48) et dans l’archipel des Mascareignes où se situe l’île de La Réunion (47); l’ISAF ne répertorie que 17 cas en Nouvelle-Calédonie et seulement 7 en Polynésie française. La faible représentation de ces deux territoires dans la base de données ISAF s’explique par un faible taux de déclaration, sachant que l’ISAF n’enregistre que les cas qui lui sont transmis. Ainsi, nous avons répertorié 54 accidents en Polynésie française de 1979 à 2001 [36], et J. Delatte en a répertorié 92 de 1980 à 2015 [12]. La Réunion est mieux représentée du fait probablement d’une meilleure déclaration, et de la fréquence élevée des attaques mortelles sur cette île, laquelle tend à favoriser cette démarche.

Les données directement collectées sur l’île de La Réunion, où ont été recensées, selon la même méthode que la nôtre, 57 attaques jusqu’à fin 2019 (G. Van Grevelynghe, communication personnelle, mars 2020), dont 50 de 1913 à 2013 [52], et 27 de 2000 à 2016 [5], pour une population de 853 659 habitants en 2017 [25] et un linéaire côtier de 210 km [29], sont proches de celles relatives à la Nouvelle-Calédonie. De même, nos données peuvent être comparées à celles relatives à la Polynésie française, relevées de façon analogue à la nôtre, et dont la population était évaluée à 268 207 habitants en 2012, et le linéaire côtier à 2 525 km [12]. Avec 67 attaques recensées, nos données semblent placer la Nouvelle-Calédonie parmi les régions du monde le plus à risque, si ce nombre d’attaques est comparé avec ceux des autres pays exposés à ce risque pour lesquels les données sont disponibles. Ainsi, il y a 4 fois plus d’attaques par habitant en Nouvelle-Calédonie qu’à La Réunion bien qu’il y ait 14 fois moins d’attaques au kilomètre de littoral. Cependant, les densités de population humaine le long du littoral diffèrent entre ces deux pays. Cette difficulté à comparer les nombres d’attaques d’un pays à l’autre est plus grande encore si on compare les ensembles insulaires français de l’Indo-Pacifique tropical avec les grands pays continentaux comme les États-Unis d’Amérique dont une moindre proportion de la population occupe la frange littorale, et pour lesquels le recueil des données de l’ISAF suit une méthode différente de la nôtre.

Ainsi que l’expose l’ISAF, la définition d’un risque d’attaque pour une population donnée suppose de rapporter le nombre d’attaques à un nombre de sujets exposés au risque de celles-ci, ce qui n’est envisageable qu’à partir d’extrapolations portant sur la population vivant sur le littoral, ou sur celle des usagers du milieu marin [26]. L’augmentation du nombre d’attaques en Nouvelle-Calédonie et celle du nombre de décès résultant de celles-ci apparaissent en cohérence avec ce qui a été observé ailleurs dans le monde depuis les deux dernières décennies du XX^e^ siècle [37, 54]. L’explication communément avancée de cette augmentation par l’augmentation de la population humaine et, par voie de conséquence, par celle du nombre d’humains exposés au risque mérite d’être discutée [16, 43]. Cette augmentation est plus rapide que celle de la population humaine [37], encore que des corrélations avec des variations du nombre d’usagers du milieu matin, tels que les baigneurs, aient été localement établies [9]. L’augmentation de la population calédonienne pourrait en partie expliquer celle du nombre d’attaques puisqu’en toute logique, le nombre d’usagers du milieu marin exposés aux contacts avec les requins est corrélé à l’augmentation de la population humaine. Au niveau mondial, les attaques et les attaques mortelles répertoriées sont également en augmentation. Cependant, les populations de requins le plus fréquemment responsables d’attaques que sont le grand requin blanc, le requin tigre et le requin bouledogue [37, 54] sont en baisse [15,17,31,40,42]. La démographie des populations locales du requin tigre et du requin bouledogue, qui sont les prédateurs le plus fréquemment impliqués dans les attaques en Nouvelle-Calédonie est, quant à elle, mal connue.

D’autres facteurs sont susceptibles d’intervenir, comme la modification de l’habitat, d’origine naturelle ou anthropique, les fluctuations d’abondance de proies, le changement climatique, la modification de la qualité (turbidité, salinité) de l’eau, ou l’augmentation locale du trafic maritime [9, 37]. Il semblerait qu’un abord de la problématique au niveau local soit plus pertinent que les considérations d’ordre général, à l’échelle du monde ou d’un pays, et que celui-ci doive prendre en compte la distribution des espèces de requins localement présentes et les facteurs environnementaux susceptibles de favoriser les attaques [38]. Ainsi, l’impact de modifications du milieu d’origine anthropique sur des populations du requin bouledogue a été décrit [53], de même que leur influence sur la fréquence des attaques attribuées à cette espèce [21, 22].

Parmi les facteurs favorisant la survenue d’une attaque de requin en Nouvelle-Calédonie, la stimulation alimentaire apparaît comme prépondérante, puisque celle-ci concerne la moitié des événements recensés. La stimulation alimentaire est tenue pour acquise dans le cas où les attaques concernent des chasseurs sous-marins. Dans d’autres cas, ont été mis en cause: les déchets de poissons des navires hauturiers à leur retour dans le lagon, la percolation d’effluves résultant d’activités de chasse à terre, l’utilisation d’engrais organiques à proximité du rivage, et plus généralement l’anthropisation du littoral, comprenant une urbanisation mal maîtrisée. Les facteurs réputés favoriser les attaques (turbidité de l’eau, nuit ou crépuscule, victime isolée) ne sont identifiés que dans un quart des cas. La recherche de facteurs explicatifs des attaques récentes aux abords immédiats de Nouméa est en cours, mais celle-ci est rendue délicate par (i) le nombre peu élevé d’attaques, qui limite la puissance de l’analyse statistique et (ii) la difficulté intrinsèque à l’évaluation de certaines données (populations de requins, facteurs environnementaux d’origine anthropique, impact des comportements humains). Enfin, l’utilisation d’un foil lors de certaines activités sportives (kitesurf, SUP foil) pourrait constituer un stimulus auditif et visuel vis-à-vis de certains grands prédateurs opportunistes tels que le requin tigre et le requin bouledogue.

La létalité des attaques en Nouvelle-Calédonie (19 %) apparaît supérieure à la moyenne mondiale, en particulier ces deux dernières décennies où elle est estimée à 7 % par l’ISAF [26]. Elle est en revanche inférieure à celle observée à La Réunion, qui, avec 57 attaques dont 23 létales, est de 40 % (G. Van Grevelynghe, communication personnelle, mars 2020), et supérieure à celle relevée en Polynésie française (nulle dans les deux séries les plus récentes) [12, 36]. Toutefois, les données de l’ISAF ne concernent que les attaques dites « non provoquées », dont sont exclues celles sur les chasseurs sous-marins, ce qui amène une discordance avec notre méthode de recueil des données. Selon A.K. Lentz et co-auteurs [30], la gravité des lésions au niveau mondial se répartit ainsi: 8 % de niveau 5, 15 % de niveau 4, 19 % de niveau 3, 17 % de niveau 2 et 42 % de niveau 1. Nos données en diffèrent essentiellement quant à la proportion des lésions de niveaux 4 et 5 et par celle de lésions de niveau 1. Ceci s’explique par une mortalité plus élevée dans notre série, les lésions les moins sévères étant en revanche moins fréquemment recensées en Nouvelle-Calédonie qu’au niveau mondial.

La proportion d’attaques en surface ou à proximité immédiate de celle-ci (78 %) ne diffère pas des données mondiales [26]. Cette caractéristique s’est affirmée par rapport à notre travail précédent, puisque les attaques en surface n’y représentaient que 56 % des cas [35]. Elle semble résulter d’un changement de la typologie des attaques, dont sont victimes une proportion croissante d’usagers évoluant en surface (cf.infra).

La saisonnalité des attaques apparaît bien établie [46]. Elle peut s’expliquer par la plus grande fréquentation humaine du lagon durant l’été austral, mais également par l’abondance des précipitations susceptibles d’alimenter les eaux côtières en sédiments et nutriments, par la température élevée de l’eau, susceptible d’influencer le comportement des requins et par le cycle reproductif de certains d’entre eux, par exemple le requin tigre et le requin bouledogue [41]. Les femelles de cette dernière espèce mettent bas dans des eaux peu profondes proches du rivage à la saison chaude [6, 31].

La prépondérance des attaques, ainsi que des attaques mortelles, attribuées en Nouvelle-Calédonie au requin tigre et au requin bouledogue, et dans lesquelles n’intervient que relativement peu le grand requin blanc, alors que le requin gris de récif y est responsable d’attaques non mortelles, pourrait refléter la distribution des espèces potentiellement vulnérantes pour l’homme dans les eaux de cet archipel. Le grand requin blanc y semble peu présent, bien qu’il y soit régulièrement observé [2,18,23,28,32,46,48,51], or cette espèce est responsable de la grande majorité des attaques dans le monde, y compris mortelles (respectivement 43 % et 42 %) [26], suivie du requin tigre (respectivement 17 % et 27 %) et du requin bouledogue (respectivement 15 % et 20 %), alors que le requin gris de récif n’apparaît que de façon marginale (< 1 %). La létalité des attaques par requin tigre en Nouvelle-Calédonie (40 %) apparaît supérieure à ce qu’elle est dans le monde (26 %) [26]; dans certains cas, l’éloignement des victimes et les délais de transport apparaissent comme des éléments de mauvais pronostic [33], mais d’autres facteurs tels que la motivation supposée des attaques (fréquence des lésions délabrantes attribuables à une finalité alimentaire) sont susceptibles d’intervenir. L’implication du requin bouledogue dans les attaques en Nouvelle-Calédonie est légèrement supérieure à la moyenne mondiale (21 % contre 15 %), en particulier dans les attaques récemment observées aux abords immédiats de Nouméa, où il est majoritaire (4 cas sur 7, soit 57 %).

La proportion de victimes immergées lors des attaques est inférieure à celle constatée lors de notre précédent travail recensant ce type d’événement jusqu’en 2009 (87,5 %) [35]. Inversement, la proportion d’adeptes des sports de glisse (kitesurf, surf, planche à voile, SUP foil) est passée de 3 % à 13,5 %, ce qui pourrait refléter une redistribution des activités de prédilection au sein des usagers de loisir du milieu marin; cette hypothèse n’a pu toutefois être étayée par une enquête sociologique, une grande majorité de sportifs exerçant, à notre connaissance, hors de tout cadre fédéral permettant un recensement via les ligues sportives. L’attaque d’une plongeuse en scaphandre autonome, survenue en 2020, est un évènement exceptionnel; c’est le premier cas répertorié en Nouvelle-Calédonie à notre connaissance.

La tendance mondiale est une nette augmentation des attaques visant les pratiquants de sports de glisse et une diminution de celles visant les plongeurs (cette catégorie incluant selon l’ISAF les plongeurs, les apnéistes, chasseurs sous-marins ou non, et les plongeurs en scaphandre autonome) [26]. La gravité des lésions et la létalité des attaques observées en Nouvelle-Calédonie ne sont pas corrélées à l’activité exercée par la victime.

Enfin, il convient de souligner que notre recueil de données n’est pas exhaustif. D’une part, un certain nombre d’attaques à l’origine de blessures de faible gravité (niveau 1) ont pu faire l’objet de soins dans des dispensaires loin de Nouméa et ont pu échapper à notre recensement du fait de l’absence de transfert des victimes sur le CHT-NC. D’autre part, la définition de l’attaque de requin que nous avons appliquée exclut 17 disparitions en mer dont le lien avec une attaque de requin ne peut être formellement prouvé en l’absence de corps et en l’absence de témoins [46]. Les 2 cas les plus récents concernent un chasseur sous-marin disparu à Maré en 2019 et un véliplanchiste disparu à Nouméa en 2020; les empreintes dentaires retrouvées sur le matériel de ces disparus signent l’intervention d’un requin de grande taille. La chronologie des faits permet de suspecter une attaque de requin létale, mais une prédation posthume ne peut être écartée.

À l’île de La Réunion, un programme d’étude des requins tigres et bouledogues, mené de 2011 à 2015 et utilisant le marquage de spécimens et le suivi de leurs déplacements par des balises côtières, a permis d’affiner la connaissance des phénomènes déterminant la présence des requins bouledogues sur la côte [45]. Parallèlement, et bien qu’il s’agisse d’espèces figurant sur la liste rouge de l’IUCN [15, 42], un programme d’abattage a été entrepris, destiné à contrôler les populations de requins tigres et bouledogues dans les zones les plus fréquentées par les usagers du milieu marin [19]. En Nouvelle-Calédonie, plusieurs campagnes d’abattage des mêmes espèces ont été menées aux abords de Nouméa à partir de 2019, alors qu’a débuté un programme de marquage et de surveillance par balises côtières dont des résultats préliminaires ont été rendus publics [4].

## Conclusion

Au total, les attaques de requins en Nouvelle-Calédonie se caractérisent par: un nombre élevé, plaçant cet ensemble insulaire parmi les endroits du monde les plus exposés à ce type de risque; une forte létalité des attaques; une augmentation du nombre absolu d’attaques comparable à la tendance observée au niveau mondial; une prédominance des attaques imputables au requin tigre, au requin bouledogue et au requin gris de récif; une prédominance du requin tigre parmi les espèces responsables d’attaques létales; une prédominance des chasseurs sous-marins parmi les victimes et possiblement une tendance à l’augmentation au fil des années de la proportion des baigneurs, nageurs et adeptes du snorkeling ainsi que des pratiquants des sports de glisse. L’apparition récente d’attaques aux abords immédiats de Nouméa, sous la dépendance de facteurs locaux, appelle une analyse détaillée.

## Remerciements

Nos plus vifs remerciements à Serge Andréfouët [3] qui nous a transmis le fond de carte utilisé pour la Figure 4 et à Yves Lefèvre pour les photos de requins reproduites avec son aimable autorisation (Figures 7, 8, 9, 10, 11, 12).

## Liens d’intérêts

Les auteurs ne déclarent aucun lien d’intérêt.

## Contribution des auteurs

Recueil des données: P.T., C.M., A-L. G., J.F.

Définition de la méthodologie: C.M., P.T., P.B.

Traitement des données: C.M., P.T., P.B.

Conception du manuscrit: C.M., P.T., P.B, M.N.

Prospection bibliographique: C.M., P.B., P.T., M.N.

Rédaction du manuscrit: C.M., P.T., P.B., A-L.G., J.F., M.N.

Conception des figures: P.B., C.M., P.T.

Analyse statistique: P.B.

Relecture et validation du manuscrit: C.M., P.T., P.B., A-L.G, J.F., M.N.
